# The effectiveness of nurse residency programs on new graduate nurses' retention: Systematic review

**DOI:** 10.1016/j.heliyon.2024.e26272

**Published:** 2024-02-17

**Authors:** Zahra Mohamed, Nathira Al-Hmaimat

**Affiliations:** a Sheikh Shakhbout Medical City, Abu Dhabi, United Arab Emirates; b Fatima College of Health Sciences, Abu Dhabi, United Arab Emirates

**Keywords:** Nurse, Graduate nurses, Residency, Internships, Nurse residency program, Retention, Turnover and retention rate

## Abstract

**Background:**

The World Health Organization estimated a need for around 6 million nurses by 2030 to meet the healthcare demand. The International Council of Nurses reported that, the impact of COVID-19 pandemic, the aging nursing workforce, and the high turnover of nurses were some of the factors that contributed to the anticipated 13 million nursing deficit. Globally, there is a worry about the high turnover with no doubt. The cost of turnover and recruitment incurred by healthcare organizations are huge which requires developing measures and interventions to address the problem. Nurse Residency Programs is a promising educational intervention for improving nurses' retention rates and reducing the impact of the shortage.

**PICO question:**

What effect does successful completion of Nurse Residency Program have on new nurses’ retention rates compared with new nurses who missed the Nurse Residency Program?

**Inclusion criteria:**

The review included articles; published in English, between 2016 and 2023 that addressed nurse retention rates, associated with Nurse Residency Program participation.

**Methods:**

The systematic review followed the PRISMA protocol. An extensive search on the Cumulative Index of Nursing and Allied Health Literature (CINAHL), MEDLINE, Academic Search Complete, and PubMed for studies published between January 2016 and March 2023 in English language. The key words, ‘graduate nurses’, ‘new graduate nurses’, ‘residency’, ‘internships and residency’, ‘NRP’, ‘retention and retention rates’. Utilizing the JBI Sumari, two reviewers screened the citations, reviewed the eligibility criteria, conducted the critical appraisal, and assessed the risk of bias and extracted data from the included studies. Cochrane Risk of Bias in Non – randomized studies of intervention (ROBINS-I) tool was used to assess risk for bias.

**Results:**

Out of 189 studies, 48 studies removed as duplicate, remaining with 141 article. After screening titles and abstracts, only 48 papers retrieved for full-text evaluation. Out of 48 research only 5 publications were included in the review. The researchers identified the methodological heterogeneity is a major factor to stop the metanalysis and keep the systematic review.

**Conclusions:**

Nurse Residency Programs showed promise as an educational intervention to cultivate well-supported, competent, and confident new nurses. These programs have the potential to improve retention rates during the initial 12 months of employment. To gain a deeper understanding of retention beyond the first year, additional randomized control trials are essential. Furthermore, there is a need to integrate standardized Nurse Residency Programs into organizational policies and clinical practice settings in the UAE.

## Teaser

Registration: Not Registered.

**Tweetable abstract:** Nurse Residency Programs are a promising educational intervention to enhance the retention of competent, and confident new nurses.

## What is already known

• In the United Arab Emirates, 96% of the healthcare professionals in Abu Dhabi are expatriate.
• The financial viability of healthcare institutions is strongly impacted by nursing turnover.
• The nurse residency programs play role in nurse retention, increased confidence and competence, and overall satisfaction.

## What this paper adds

• A dedicated and trained preceptors are needed to support the Nurse residents throughout the nurse residency program.
• All nurse residency programs must have at least 12-month transitional periods, as the retention rate is lower for shorter programs.
• Consider the program as workforce development and retention strategy in the organization strategic plan.

## 1 Introduction

The International Council of Nurses estimated 13 million of nursing shortage due to several factors such as the aging nursing workforce, impact of COVID-19, and the nurses leaving the profession [1]. In addition, The World Health Organization estimates that about 6 million nurses will be needed by 2030 to meet global needs [1]. Nursing staff forms most of the healthcare workforce. Wherein, the high turnover folded the shortage of nurses that caused a challenge to many countries around the globe [2]. Both the shortage and turnover trigger a negative impact on providing high-quality of care [2]. Research has shown that nurses are under extraordinary overwork, inadequate resources, stress, and emotional strain, all of those are factors leading experienced nurses leave the profession [1,2]. In addition, the scarcity of nurses around the world has been a problem for many nations, which has increased strain on the existing workforce and had a negative influence on staff wellbeing and patient care [2].

The financial viability of healthcare institutions is strongly impacted by nursing turnover. Along with expenditures, the phenomenon's effects also healthcare accessibility [3]. For instance, Australia, Canada, and the United States each have yearly nursing turnover rates of about 15%, 20%, and 27%, respectively [3]. In Jordan, the anticipated turnover is comparatively higher at 32%. Interestingly, new nursing graduates' turnover rates seem to be exceptionally high [3]. The United Sates may face serious public health emergency, if its healthcare system keeps losing senior registered nurses [3]. Poor patient outcomes are more likely due to a shortage of skilled nurses, which can directly lead to higher hospital expenses [3]. More significantly, a lack of nurses has been connected to higher mortality rates, staff violence, accidents and injuries, cross-infection, and unfavourable postoperative outcomes [3].

Considering the cost and financial implications of the turnover, it has been identified that new graduates have higher turnover costs higher than experienced nurses due to the amount of dedicated training hours, the need of experienced educators and competent preceptors [7,8]. Research has shown that new hire's recruitment and on boarding process expenses equal one and a half times the annual compensation of an experienced nurse, therefore, loosing those new nurses after the huge investments on them adds burden to healthcare organization [7,8].

Researchers argue that the world's disastrous shortage of nurses was completely predictable and preventable [1]. The negative impact the nurse shortage has on the ability of the world's healthcare organization to provide equitable and accessible healthcare, is particularly huge [1]. Healthcare organizations are adopting several retention measures to overcome the shortage, reduce new nurses' burnout and to ensure that patient care is optimal. Turnover and satisfaction issues are believed to be preventable crisis through meaningful and proactive solutions. Research highly supported investing on the new nurse graduates. Healthcare leaders and policy makers can mitigate the challenges facing both new nurses and seasoned nurses through evidence-based initiatives that aim at empowering and foster resilience [4].

Nurse Residency Programs (NRP) are structured and comprehensives program that facilitate smooth transition of new graduate nurses from being student into an independent healthcare professional who can safely and competently take care of a patient [6]. NRP helps new graduates familiarize with real-world experience, skills and competencies needed in a supportive environment that allows gradual integration and learner paced approach into the healthcare system [6].

Foreign nurses represent a significant portion of the essential healthcare workforce in Gulf countries like Saudi Arabia and the United Arab Emirates (UAE) [3,5]. Consequently, the increasing turnover rates among foreign nurses in healthcare facilities nationwide pose a substantial problem. In the capital of the UAE, Abu Dhabi, 96% of healthcare professionals come from various countries, and 82% of the city's population consists of expatriates [5]. Similarly, in the Emirate of Dubai, the second-largest in the UAE, 90% of healthcare workers are expatriates, and 91% of the population is made up of immigrants [5]. Furthermore, a substantial portion of healthcare workers in neighboring Gulf countries are also expatriates, creating a challenge for the healthcare system due to their high turnover rates [3,5]. It's worth noting that there is no reported data on turnover rates in both countries, which could be valuable for predicting future trends and comparing with other nations.

The effectiveness of these programs should be assessed in terms of their impact on nurse retention, boosting self-assurance and skills, and enhancing overall job satisfaction. Therefore, the researcher aims to conduct a systematic review of the effectiveness of the Nurse Residency program on retention, which shall add to the current knowledge on the topic and guide policy making in the UAE.

## 2 Method

The systematic review followed the Preferred Reporting Items for Systematic Reviews PRISMA checklist (REFERENCE- MOHER). There were two reviewers utilizing the same databases, which are the Cumulative Index of Nursing and Allied Health Literature (CINAHL), MEDLINE, PubMed, and Academic Search Complete using the key words: graduate nurses, new graduate nurses, residency, internships, and residency, NRP, retention and retention rate. The Boolean operator “AND” or “OR” were employed to connect each keyword to the next in the advance search tabs, which helped to narrow the search results.

### 2.1 Information sources

Different databases such as CINAHL, MEDLINE and PubMed, Academic Search Complete were conducted between 3rd January to February 28, 2023. Several studies were not found with full text and the librarian was contacted to obtain the digital full access article. There was one systematic review, which was in Korean language, and no translation was found even after seeking the librarian support.

### 2.2 Search of baseline systematic review

The researchers conducted an initial search through the PRISMA registration website. The verification of registration for the same systematic review is not found. However, this systematic review is not registered at PRISMA for the fact that, the review and risk assessment were completed before the time of registration.

### 2.3 PICO question

This systematic review aims to examine the impact and the effectiveness of Nurse Residency Program on the new graduate nurses' retention rates. The PICO question: What effect does successful completion of Nurse Residency Program (NRP) have on new nurses’ retention rates compared with new nurses who did not participate in an NRP?

The Key words used in the review are; graduate nurses, new graduate nurses, residency, internships, NRP, retention, rates.

### 2.4 Inclusion criteria & exclusion criteria

The eligibility criteria encompassed the following:

a. Studies concentrating on participants who were newly graduated nurses entering healthcare organizations as new hires with fewer than 12 years of experience.
b. Studies selecting Nurse Residency Programs as the intervention or the phenomenon of interest.
c. Studies that examined the outcomes related to the retention rates of newly graduated nurses who had successfully completed the Nursing Residency Program.

All empirical research studies targeting new nurses and their retention rate were included in this review. The included research methods followed the quantitative experimental and non-experimental studies. This review excluded qualitative, non-comparative studies and program evaluation and other articles which were theoretical research, literature reviews, quality improvements, conference abstracts and editorials. Inclusion criteria and Exclusion criteria were listed in Table 1.

**Table-1:** Inclusion and exclusion criteria.

INCLUSION CRITERIA	EXCLUSION CRITERIA
ARTICLES PUBLISHED BETWEEN 2016 AND 2022.	Articles addressing program development and curriculum without a discussion of retention rates.
ARTICLES ADDRESSED NURSE RETENTION RATES LINKED TO THE EFFECT OF NRPS.	Articles focusing on preceptor-only orientation processes,
ARTICLES PUBLISHED IN ENGLISH.	Articles addressing NRPs implementations

### 2.5 Search strategy

The search strategy followed the PICO question where in the study population was the new graduate nurse, the intervention was nurse residency program, and the outcome was the retention rate. In addition, terms and keywords derived from initial scoping search and Mesh terms were used such as nurse, Education, training, orientation, turnover, competency, skills, graduate. See Table 2: Search strategy. The PredicTER Software was utilized to estimate the time frames for the completion of the systematic review.

**Table 2:** Search strategy.

DATABASE	SEARCH STRATEGY	NUMBER OF ARTICLES EXPORTED TO ENDNOTE	SEARCH CRITERIA
CINAHL via Ovid	S1: graduate nurses OR new graduate nurses S2: residency OR internships OR residency OR NRP S3: Retention rate OR Retention S4: S1 AND S2 and S3	95	English Language Published between January 2016 and February 2023
Academic search		47	
Medline		40	
PubMed		7	
Total	189		

First stage search, a limited set of key words were used for the search to find possibly relevant studies. The initial identified studies were reviewed for new key words to allow for an in-depth search.

In stage two, the references list of papers that were identified were reviewed for any pertinent new references. Additionally, a search for researchers whose names were repeated in several articles and were known to have done research on the review topic objective were reviewed.

### 2.6 Study selection

**Fig. 1:**
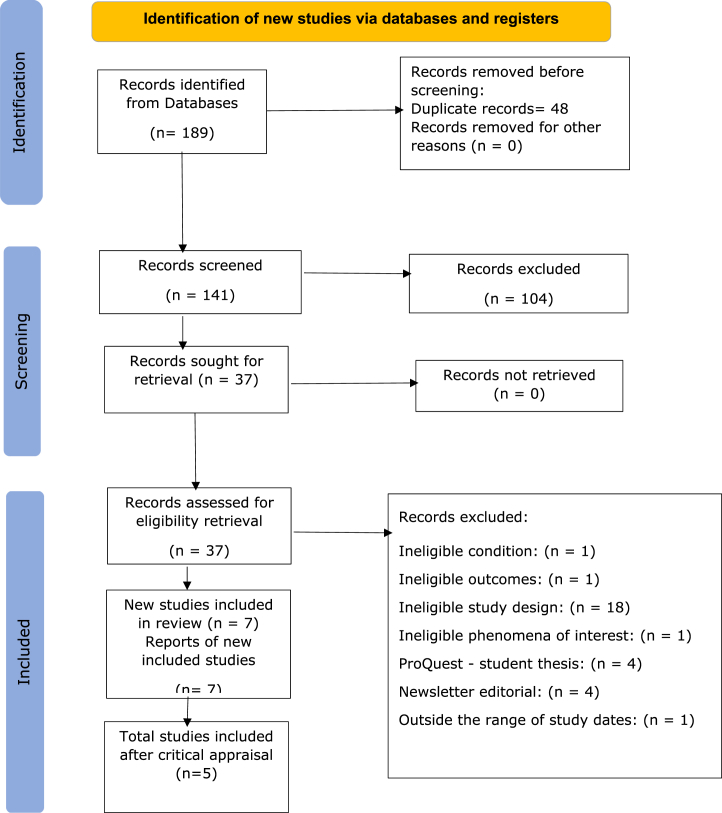
Prisma diagram.

All identified articles were compiled and uploaded to the EndNote software, the identified missing information such as the authors, year of publication and the Full text of the article were entered manually. This step was followed by importing the articles after removing the duplicate into the JBI SUMARI [10]. Screening of the imported articles was done at three levels; initial with title and abstract screening followed by full text screening. The full text studies were evaluated against the inclusion and exclusion criteria. The appropriate JBI Sumari critical appraisal tool was used to determine the quality of the studies by the two researchers. The PRISMA flow diagram is provided (see Fig. 1) for the number of studies and the process of inclusion and exclusion (Table 2, Table 5, Table 6, Table 7).

**Table 3-a:** Characteristics of Included Studies - Longitudinal Prospective Study-Case series.

STUDY	COUNTRY	SETTING/CONTEXT	PARTICIPANT CHARACTERISTICS	GROUPS	OUTCOMES MEASURED	DESCRIPTION OF MAIN RESULTS
FAILLA KR, ECOFF L, STICHLER JF, PELLETIER LR. 2021.	Southern California, US	Five Hospitals of Sharp Healthcare Hospitals	Participants Age Range 22–54 Female = 86 % Male = 14%	The Sample Included 117 NGNRS Across The 5 Hospitals.	**Retention Rate**	For The 117 NGNR Retention Rate At 12 Months Was 85% Since Inception of the System NRP, the 1-Year Retention is Greater Than 96% (N = 241)

**Table 3-b:** Characteristics of included studies - quasi-experimental study form.

STUDY	COUNTRY	SETTING/CONTEXT	PARTICIPANT CHARACTERISTICS	GROUPS	OUTCOMES MEASURED	MAIN DESCRIPTION OF RESULTS
MILLER CM, MEYER K, RIEMANN LA, CARTER BT, BRANT JM. 2023.	Northwest Region, United States	3 Times Magnet Designated Hospital	Newly Employed Nurses. Female = 87.7%, Male = 12.3% Mean Age 28.5 Years	Seven Cohorts. Total = 106, Nurse Resident = 77, Control Group = 29	Readiness For Practice, Improved **Nurse Retention**, And Higher Job Satisfaction Compared with Standard Orientation.	The Retention Rate of Nurse Residents Was 77.27% for the Three Cohorts Enrolled In 2016. 90.20% For the Three Cohorts Enrolled In 2017. And 89.71% For the Cohort Enrolled In 2018.
SALMOND SW, CADMUS E, BLACK KK, BOHNARCZYK N, HASSLER L. 2017.	New Jersey, US	Long-Term Care Facility	Newly Hired Graduate RN Female 75%, Male 11.1%	Two Cohorts, N = 36	**Retention**, Nurse Resident Confidence, Clinical Practice	The current nurse residency program experienced an **86%** retention rate through the first 12 months.
PILLAI S, MANISTER NN, COPPOLO MT, DUCEY MS, MCMANUS-PENZERO J. 2018.	Northeastern, US	305 Bed, Acute Care, Tertiary Hospital Magnet Designated Hospital	Age Range 21–62 Female 88.3%, Male 1.7% Newly Licensed Nurses with Less Than One-Year Experience	20 Cohorts N = 241 Nurse Residents	**Retention Rate**	Out of the 241-nurse resident, 212 nurses were retained after one year with retention rate of **96%** the gems home grown program retention rates were higher than vendor based programs.

**Table 3-c:** Characteristics of Included Studies - Experimental retrospective pre-post test.

STUDY	COUNTRY	SETTING/CONTEXT	PARTICIPANT CHARACTERISTICS	GROUP A DESCRIPTION AND SAMPLE	GROUP B DESCRIPTION AND SAMPLE	EXPOSURES/VARIABLES MEASURED	DESCRIPTION OF MAIN RESULTS
WOLFORD J. 2019.	Southern region of the US	Healthcare system comprised of five hospitals.	age range 22–59 years with mean age 29.7.81.7% female and 18.3% male nursing experience ranged from 16 to 32 months.	Control group = 791 newly licensed nurses hired prior to the NRP	Intervention group = 232 newly licensed nurses	Turnover rate, retention, engagement, and program ROI.	Significant decrease in turnover for the group who participated in the NRP. 14% of turnover among the control group with a retention rate of = 86%, 3.5% of turnover from intervention group which is 96.5%.

**Table 4-a:** Critical appraisal of eligible analytical cross-sectional study.

CITATION	Q1	Q2	Q3	Q4	Q5	Q6	Q7	Q8
CADMUS E, ROBERTS ML. 2022.	N	N	N	N	N	N	Y	Y
%	0.0	0.0	0.0	0.0	0.0	0.0	100.0	100.0

**Table 4-b:** Critical appraisal of eligible case control study.

CITATION	Q1	Q2	Q3	Q4	Q5	Q6	Q7	Q8	Q9	Q10
WOLFORD J. 2019.	Y	Y	Y	Y	Y	N	N	Y	Y	Y
%	100.0	100.0	100.0	100.0	100.0	0.0	0.0	100.0	100.0	100.0

**Table 4-c:** Critical appraisal of eligible case series.

CITATION	Q1	Q2	Q3	Q4	Q5	Q6	Q7	Q8	Q9	Q10
PELLETIER LR, VINCENT C, WOODS L, ODELL C, STICHLER JF. 2019.	Y	Y	Y	Y	N	Y	Y	Y	Y	Y
FAILLA KR, ECOFF L, STICHLER JF, PELLETIER LR. 2021.	Y	Y	Y	Y	Y	Y	Y	Y	Y	Y
%	100.0	100.0	100.0	100.0	50.0	100.0	100.0	100.0	100.0	100.0

**Table 4-d:** Critical appraisal of eligible quasi-experimental study.

CITATION	Q1	Q2	Q3	Q4	Q5	Q6	Q7	Q8	Q9
MILLER CM, MEYER K, RIEMANN LA, CARTER BT, BRANT JM. 2023.	Y	Y	Y	Y	Y	Y	Y	Y	Y
SALMOND SW, CADMUS E, BLACK KK, BOHNARCZYK N, HASSLER L. 2017.	Y	Y	Y	Y	Y	Y	Y	Y	Y
PILLAI S, MANISTER NN, COPPOLO MT, DUCEY MS, MCMANUS-PENZERO J. 2018.	Y	N	N	N	N	Y	Y	Y	Y
%	100.0	66.66	66.66	66.66	66.66	100.0	100.0	100.0	100.0

**Table 5:** Risk of Bias in Non – randomized studies of intervention (ROBINS-I) tool.

STUDY	CONFOUNDING	SELECTION OF PARTICIPANTS	CLASSIFICATION OF INTERVENTION	DEVIATION FROM INTENDED INTERVENTIONS	MISSING DATA	MEASUREMENT OF OUTCOMES	SELECTION OF REPORTED RESULTS	OVERALL
MILLER ET AL. 2023	Mean age was 28.5 years Difference in the level of education and nursing degrees. Different units and leadership Moderate risk	Convenient sampling Moderate risk No randomization,	Low risk	Low risk	Several nurses did not complete the measures, resulting in a lower sample size Moderate risk	Low risk	Low risk	Low risk
SALMOND ET AL. 2017	Age range was very high 21–49 however they tried to control by an eligible inclusion criterion by having one year of experience after graduation. Difference in the level of education of the participants Moderate risk	Nonrandomized convenient sample Differences in the selection criteria ta from cohort 1 and cohort 2 Moderate risk	Low risk	Low risk	They have difficulty in collecting data for example Several facilities were not able to provide the data on retention. Moderate risk	Low risk	Low risk	The researchers tried to have the study as mixed methodology to support as the most effective mean of putting data into context. Moderate risk
FAILLA ET AL 2021	Age range 22–54 Less than 1 year Different speciality area experience, RN license Moderate risk	Nonrandomized convenient sample Moderate risk	Low risk	Low risk	Low risk	Low risk	Low risk	Low risk
WOLFORD J. 2019.	Age range is huge 22-59 Did not mention the education level BSN vs Diploma. Moderate risk	Convenient sampling as it is a retrospective. Moderate risk	Low risk	Low risk	Low risk	Low risk	Low risk	Low risk
PILLAI ET AL 2018	Age range is huge 21-62 Different degree and educational level (Diploma, associate degree, and BSN) High risk	Convenient sampling Moderate risk	Low risk	No information	No information	Low risk		Moderate risk

**Table 6:** Excluded studies.

Studies Excluded on Full Text	REASON FOR EXCLUSION
ACKERSON K, STILES KA. VALUE OF NURSE RESIDENCY PROGRAMS IN RETAINING NEW GRADUATE NURSES AND THEIR POTENTIAL EFFECT ON THE NURSING SHORTAGE. J CONTIN EDUC NURS. 2018; 49 (6):282–8.	Ineligible study design
ADATARA P, BONI GS. NEW GRADUATE NURSES’ EXPERIENCES AND CHALLENGES DURING A ONE-YEAR CLINICAL ROTATION PROGRAMME IN THE VOLTA REGION OF GHANA: IMPLICATIONS FOR NURSING MANAGEMENT AND NURSING WORKFORCE RETENTION. BIOMED RESEARCH INTERNATIONAL [INTERNET]. 2022; 2022:1–9. AVAILABLE FROM: HTTPS://EZPROXY.UOW.EDU.AU/LOGIN?URL=HTTPS://SEARCH.EBSCOHOST.COM/LOGIN.ASPX?DIRECT=TRUE&DB=RZH&AN=161015034&SITE=EHOST-LIVE	Ineligible study design
AFRICA LM. TRANSITION TO PRACTICE PROGRAMS: EFFECTIVE SOLUTIONS TO ACHIEVING STRATEGIC STAFFING IN TODAY’S HEALTHCARE SYSTEMS. NURSING ECONOMIC$ [INTERNET]. 2017; 35 (4):178–83. AVAILABLE FROM: HTTPS://EZPROXY.UOW.EDU.AU/LOGIN?URL=HTTPS://SEARCH.EBSCOHOST.COM/LOGIN.ASPX?DIRECT=TRUE&DB=RZH&AN=124650130&SITE=EHOST-LIVE	Duplicate study
AFRICA LM. TRANSITION TO PRACTICE PROGRAMS: EFFECTIVE SOLUTIONS TO ACHIEVING STRATEGIC STAFFING IN TODAY’S HEALTHCARE SYSTEMS. NURSING ECONOMIC$ [INTERNET]. 2017; 35 (4):178–83. AVAILABLE FROM: HTTPS://EZPROXY.UOW.EDU.AU/LOGIN?URL=HTTPS://SEARCH.EBSCOHOST.COM/LOGIN.ASPX?DIRECT=TRUE&DB=RZH&AN=124650130&SITE=EHOST-LIVE	Ineligible study design
ASBER SR. RETENTION OUTCOMES OF NEW GRADUATE NURSE RESIDENCY PROGRAMS: AN INTEGRATIVE REVIEW. JONA: THE JOURNAL OF NURSING ADMINISTRATION [INTERNET]. 2019 SEP; 49 (9):435. AVAILABLE FROM: HTTPS://EZPROXY.UOW.EDU.AU/LOGIN?URL=HTTPS://SEARCH.EBSCOHOST.COM/LOGIN.ASPX?DIRECT=TRUE&DB=A9H&AN=138460459&SITE=EHOST-LIVE	Ineligible study design
BLEVINS SH. NURSE RESIDENCY PROGRAMS: SUPPORTING NURSE GRADUATES. MEDSURG NURSING [INTERNET]. 2016; 25 (5):367–8. AVAILABLE FROM: HTTPS://EZPROXY.UOW.EDU.AU/LOGIN?URL=HTTPS://SEARCH.EBSCOHOST.COM/LOGIN.ASPX?DIRECT=TRUE&DB=RZH&AN=118640447&SITE=EHOST-LIVE	Ineligible study design
CADMUS E, ROBERTS ML, OLSON E. CREATING A STATEWIDE NURSE RESIDENCY: USING AN APPRENTICESHIP MODEL. JONA: THE JOURNAL OF NURSING ADMINISTRATION [INTERNET]. 2021; 51 (11):568–72. AVAILABLE FROM: HTTPS://EZPROXY.UOW.EDU.AU/LOGIN?URL=HTTPS://SEARCH.EBSCOHOST.COM/LOGIN.ASPX?DIRECT=TRUE&DB=RZH&AN=153247702&SITE=EHOST-LIVE	Ineligible study design
CADMUS E, ROBERTS ML. FIRST YEAR OUTCOMES: PROGRAM EVALUATION OF A STATEWIDE NURSE RESIDENCY PROGRAM. JONA: THE JOURNAL OF NURSING ADMINISTRATION [INTERNET]. 2022; 52 (12):672–8. AVAILABLE FROM: HTTPS://EZPROXY.UOW.EDU.AU/LOGIN?URL=HTTPS://SEARCH.EBSCOHOST.COM/LOGIN.ASPX?DIRECT=TRUE&DB=A9H&AN=160329253&SITE=EHOST-LIVE	Duplicate study
CADMUS E, SALMOND SW, HASSLER LJ, BLACK K, BOHNARCZYK N. CREATING A LONG-TERM CARE NEW NURSE RESIDENCY MODEL. JOURNAL OF CONTINUING EDUCATION IN NURSING [INTERNET]. 2016; 47 (5):234–40. AVAILABLE FROM: HTTPS://EZPROXY.UOW.EDU.AU/LOGIN?URL=HTTPS://SEARCH.EBSCOHOST.COM/LOGIN.ASPX?DIRECT=TRUE&DB=RZH&AN=115128113&SITE=EHOST-LIVE	ProQuest - student thesis
CAPPEL CA. NURSE RESIDENCY PROGRAM: A COMPARISON OF OUTCOME MEASURES FROM 2 YEARS OF DATA FROM BSN NURSE RESIDENT GRADUATES. NURSE RESIDENCY PROGRAM: A COMPARISON OF OUTCOME MEASURES FROM 2 YEARS OF DATA FROM BSN NURSE RESIDENT GRADUATES [INTERNET]. 2016; 1–1. AVAILABLE FROM: HTTPS://EZPROXY.UOW.EDU.AU/LOGIN?URL=HTTPS://SEARCH.EBSCOHOST.COM/LOGIN.ASPX?DIRECT=TRUE&DB=RZH&AN=123313487&SITE=EHOST-LIVE	ProQuest - student thesis
CRIMLISK JT, GRANDE MM, KRISCIUNAS GP, COSTELLO KV, FERNANDES EG, GRIFFIN M. NURSE RESIDENCY PROGRAM DESIGNED FOR A LARGE COHORT OF NEW GRADUATE NURSES: IMPLEMENTATION AND OUTCOMES. MEDSURG NURSING: OFFICIAL JOURNAL OF THE ACADEMY OF MEDICAL-SURGICAL NURSES [INTERNET]. 2017; 26 (2):83. AVAILABLE FROM: HTTPS://EZPROXY.UOW.EDU.AU/LOGIN?URL=HTTPS://SEARCH.EBSCOHOST.COM/LOGIN.ASPX?DIRECT=TRUE&DB=MNH&AN=30304587&SITE=EHOST-LIVE	Ineligible study design
CRIMLISK JT, GRANDE MM, KRISCIUNAS GP, COSTELLO KV, FERNANDES EG, GRIFFIN M. NURSE RESIDENCY PROGRAM DESIGNED FOR A LARGE COHORT OF NEW GRADUATE NURSES: IMPLEMENTATION AND OUTCOMES. MEDSURG NURSING: OFFICIAL JOURNAL OF THE ACADEMY OF MEDICAL-SURGICAL NURSES [INTERNET]. 2017; 26 (2):83. AVAILABLE FROM: HTTPS://EZPROXY.UOW.EDU.AU/LOGIN?URL=HTTPS://SEARCH.EBSCOHOST.COM/LOGIN.ASPX?DIRECT=TRUE&DB=MNH&AN=30304587&SITE=EHOST-LIVE	Duplicate study
CRIMLISK JT. NURSE RESIDENCY PROGRAM DESIGNED FOR A LARGE COHORT OF NEW GRADUATE NURSES: IMPLEMENTATION AND OUTCOMES. MEDSURG NURSING [INTERNET]. 2017; 26 (2):83–104. AVAILABLE FROM: HTTPS://EZPROXY.UOW.EDU.AU/LOGIN?URL=HTTPS://SEARCH.EBSCOHOST.COM/LOGIN.ASPX?DIRECT=TRUE&DB=RZH&AN=122315808&SITE=EHOST-LIVE	Duplicate study
ECKERSON CM. THE IMPACT OF NURSE RESIDENCY PROGRAMS IN THE UNITED STATES ON IMPROVING RETENTION AND SATISFACTION OF NEW NURSE HIRES: AN EVIDENCE-BASED LITERATURE REVIEW. NURSE EDUC TODAY. 2018; 71:84–90.	Ineligible study design
FURLONG MP. A NURSE RESIDENCY PROGRAM AND THE RETENTION OF GRADUATE NURSES INTO CLINICAL PRACTICE: A DESCRIPTIVE QUANTITATIVE RESEARCH STUDY. NURSE RESIDENCY PROGRAM & THE RETENTION OF GRADUATE NURSES INTO CLINICAL PRACTICE: A DESCRIPTIVE QUANTITATIVE RESEARCH STUDY [INTERNET]. 2016; 1–1. AVAILABLE FROM: HTTPS://EZPROXY.UOW.EDU.AU/LOGIN?URL=HTTPS://SEARCH.EBSCOHOST.COM/LOGIN.ASPX?DIRECT=TRUE&DB=RZH&AN=124411326&SITE=EHOST-LIVE	ProQuest - student thesis
GORDON SC, FRANCE NEM. QUIN COUNCIL: ENTRY-INTO-PRACTICE SUPPORT PROGRAMS. FLORIDA NURSE [INTERNET]. 2017; 65 (3):14–14. AVAILABLE FROM: HTTPS://EZPROXY.UOW.EDU.AU/LOGIN?URL=HTTPS://SEARCH.EBSCOHOST.COM/LOGIN.ASPX?DIRECT=TRUE&DB=RZH&AN=125076062&SITE=EHOST-LIVE	Newsletter editorial
GORDON SC, FRANCE NEM. QUIN COUNCIL: ENTRY-INTO-PRACTICE SUPPORT PROGRAMS. FLORIDA NURSE [INTERNET]. 2017; 65 (3):14–14. AVAILABLE FROM: HTTPS://EZPROXY.UOW.EDU.AU/LOGIN?URL=HTTPS://SEARCH.EBSCOHOST.COM/LOGIN.ASPX?DIRECT=TRUE&DB=RZH&AN=125076062&SITE=EHOST-LIVE	Newsletter editorial
HERNANDEZ SHA, FRANCIS MA, WINN D. EMPLOYMENT AND RETENTION OF NURSES WHO COMPLETED AN INTERNSHIP AND RESIDENCY PROGRAM. J CONTIN EDUC NURS. 2020; 51 (11):504–8.	Ineligible phenomena of interest
HERNANDEZ SHA, FRANCIS MA, WINN D. EMPLOYMENT AND RETENTION OF NURSES WHO COMPLETED AN INTERNSHIP AND RESIDENCY PROGRAM. JOURNAL OF CONTINUING EDUCATION IN NURSING [INTERNET]. 2020; 51 (11):504–8. AVAILABLE FROM: HTTPS://EZPROXY.UOW.EDU.AU/LOGIN?URL=HTTPS://SEARCH.EBSCOHOST.COM/LOGIN.ASPX?DIRECT=TRUE&DB=RZH&AN=146628221&SITE=EHOST-LIVE	Duplicate study
KONERI L, GREEN A, GILDER RE. TOUCHPOINTS: A BUSINESS STRATEGY TO RETAIN NEW GRADUATE NURSES. JONA: THE JOURNAL OF NURSING ADMINISTRATION [INTERNET]. 2021; 51 (7/8):401–8. AVAILABLE FROM: HTTPS://EZPROXY.UOW.EDU.AU/LOGIN?URL=HTTPS://SEARCH.EBSCOHOST.COM/LOGIN.ASPX?DIRECT=TRUE&DB=RZH&AN=151993138&SITE=EHOST-LIVE	Ineligible study design
KRAM SL, WILSON J. NURSE RESIDENCY PROGRAM ATTRACTS AND RETAINS NOVICE NURSES. NURSING [INTERNET]. 2016; 46 (2):15–6. AVAILABLE FROM: HTTPS://EZPROXY.UOW.EDU.AU/LOGIN?URL=HTTPS://SEARCH.EBSCOHOST.COM/LOGIN.ASPX?DIRECT=TRUE&DB=RZH&AN=112362407&SITE=EHOST-LIVE	Ineligible study design
LESHER BB, WITT JM, WOODARD RM, HABERYAN AB. NEW GRADUATE RN PERINATAL INTERNSHIP. J CONTIN EDUC NURS. 2021; 52 (1):47–52.	Duplicate study
LINSCHEID NJ, BELL DJ. THE JOURNEY OF A HOME CARE NURSE RESIDENCY PROGRAM. HOME HEALTHCARE NOW [INTERNET]. 2021; 39 (2):99–106. AVAILABLE FROM: HTTPS://EZPROXY.UOW.EDU.AU/LOGIN?URL=HTTPS://SEARCH.EBSCOHOST.COM/LOGIN.ASPX?DIRECT=TRUE&DB=MNH&AN=33662968&SITE=EHOST-LIVE	Ineligible study design
MILLER CM, MEYER K, RIEMANN LA, CARTER BT, BRANT JM. TRANSITION INTO PRACTICE: OUTCOMES OF A NURSE RESIDENCY PROGRAM. J CONTIN EDUC NURS. 2023; 54 (1):32–9.	Duplicate study
PELLETIER LR, VINCENT C, WOODS L, ODELL C, STICHLER JF. EFFECTIVENESS OF A PSYCHIATRICÂ€“MENTAL HEALTH NURSE RESIDENCY PROGRAM ON RETENTION. JOURNAL OF THE AMERICAN PSYCHIATRIC NURSES ASSOCIATION [INTERNET]. 2019; 25 (1):66–75. AVAILABLE FROM: HTTPS://EZPROXY.UOW.EDU.AU/LOGIN?URL=HTTPS://SEARCH.EBSCOHOST.COM/LOGIN.ASPX?DIRECT=TRUE&DB=RZH&AN=134281966&SITE=EHOST-LIVE	Duplicate study
PERRON T, GASCOYNE M, KALLAKAVUMKAL T, KELLY M, DEMAGISTRIS N. EFFECTIVENESS OF NURSE RESIDENCY PROGRAMS. JOURNAL OF NURSING PRACTICE APPLICATIONS & REVIEWS OF RESEARCH [INTERNET]. 2019; 9 (2):48–52. AVAILABLE FROM: HTTPS://EZPROXY.UOW.EDU.AU/LOGIN?URL=HTTPS://SEARCH.EBSCOHOST.COM/LOGIN.ASPX?DIRECT=TRUE&DB=RZH&AN=138957438&SITE=EHOST-LIVE	Outside the range of study dates
PERRON T, GASCOYNE M, KALLAKAVUMKAL T, KELLY M, DEMAGISTRIS N. EFFECTIVENESS OF NURSE RESIDENCY PROGRAMS. JOURNAL OF NURSING PRACTICE APPLICATIONS & REVIEWS OF RESEARCH [INTERNET]. 2020; 10 (1):48–52. AVAILABLE FROM: HTTPS://EZPROXY.UOW.EDU.AU/LOGIN?URL=HTTPS://SEARCH.EBSCOHOST.COM/LOGIN.ASPX?DIRECT=TRUE&DB=RZH&AN=143154695&SITE=EHOST-LIVE	Ineligible study design
POTTINGER L. CRITICAL CARE TRANSITION PROGRAM FOR NEWLY GRADUATED NURSES. CANADIAN JOURNAL OF CARDIOVASCULAR NURSING [INTERNET]. 2016; 26 (3):5–5. AVAILABLE FROM: HTTPS://EZPROXY.UOW.EDU.AU/LOGIN?URL=HTTPS://SEARCH.EBSCOHOST.COM/LOGIN.ASPX?DIRECT=TRUE&DB=RZH&AN=117367976&SITE=EHOST-LIVE	Duplicate study
POTTINGER L. CRITICAL CARE TRANSITION PROGRAM FOR NEWLY GRADUATED NURSES. CANADIAN JOURNAL OF CARDIOVASCULAR NURSING [INTERNET]. 2016; 26 (3):5–5. AVAILABLE FROM: HTTPS://EZPROXY.UOW.EDU.AU/LOGIN?URL=HTTPS://SEARCH.EBSCOHOST.COM/LOGIN.ASPX?DIRECT=TRUE&DB=RZH&AN=117367976&SITE=EHOST-LIVE	Newsletter editorial
RUSH KL, JANKE R, DUCHSCHER JE, PHILLIPS R, KAUR S. BEST PRACTICES OF FORMAL NEW GRADUATE TRANSITION PROGRAMS: AN INTEGRATIVE REVIEW. INT J NURS STUD. 2019; 94:139–58.	Ineligible study design
SHINNERS J, AFRICA L, MALLORY C, DURHAM H. VERSANT’S NURSE RESIDENCY PROGRAM: A RETROSPECTIVE REVIEW. NURSING ECONOMIC$ [INTERNET]. 2021; 39 (5):239–46. AVAILABLE FROM: HTTPS://EZPROXY.UOW.EDU.AU/LOGIN?URL=HTTPS://SEARCH.EBSCOHOST.COM/LOGIN.ASPX?DIRECT=TRUE&DB=RZH&AN=152989847&SITE=EHOST-LIVE	Ineligible study design
SHINNERS J, AFRICA L, MALLORY C, DURHAM H. VERSANT’S NURSE RESIDENCY PROGRAM: A RETROSPECTIVE REVIEW. NURSING ECONOMIC$ [INTERNET]. 2021; 39 (5):239–46. AVAILABLE FROM: HTTPS://EZPROXY.UOW.EDU.AU/LOGIN?URL=HTTPS://SEARCH.EBSCOHOST.COM/LOGIN.ASPX?DIRECT=TRUE&DB=RZH&AN=152989847&SITE=EHOST-LIVE	Ineligible study design
SMITH JB, RUBINSON D, ECHTENKAMP D, BROSTOFF M, MCCARTHY AM. EXPLORING THE STRUCTURE AND CONTENT OF HOSPITAL-BASED PEDIATRIC NURSE RESIDENCY PROGRAMS. JOURNAL OF PEDIATRIC NURSING [INTERNET]. 2016; 31 (2):187–95. AVAILABLE FROM: HTTPS://EZPROXY.UOW.EDU.AU/LOGIN?URL=HTTPS://SEARCH.EBSCOHOST.COM/LOGIN.ASPX?DIRECT=TRUE&DB=RZH&AN=113280973&SITE=EHOST-LIVE	Ineligible condition
THEW J. LTC NURSE RESIDENCY RESULTS IN 86% RETENTION RATE. PPS ALERT FOR LONG-TERM CARE [INTERNET]. 2018; 21 (8):4–5. AVAILABLE FROM: HTTPS://EZPROXY.UOW.EDU.AU/LOGIN?URL=HTTPS://SEARCH.EBSCOHOST.COM/LOGIN.ASPX?DIRECT=TRUE&DB=RZH&AN=131246355&SITE=EHOST-LIVE	Newsletter editorial
TORRES A. IMPROVEMENT IN THE RETENTION OF GRADUATE NURSES IN A LONG-TERM ACUTE CARE HOSPITAL. IMPROVEMENT IN THE RETENTION OF GRADUATE NURSES IN A LONG-TERM ACUTE CARE HOSPITAL [INTERNET]. 2018; 1–1. AVAILABLE FROM: HTTPS://EZPROXY.UOW.EDU.AU/LOGIN?URL=HTTPS://SEARCH.EBSCOHOST.COM/LOGIN.ASPX?DIRECT=TRUE&DB=RZH&AN=131796599&SITE=EHOST-LIVE	ProQuest - student thesis
TYNDALL DE, SCOTT ES, JONES LR, COOK KJ. CHANGING NEW GRADUATE NURSE PROFILES AND RETENTION RECOMMENDATIONS FOR NURSE LEADERS. J NURS ADM. 2019; 49 (2):93–8.	Ineligible outcomes
WALSH AL. NURSE RESIDENCY PROGRAMS AND THE BENEFITS FOR NEW GRADUATE NURSES. PEDIATRIC NURSING [INTERNET]. 2018; 44 (6):275–9. AVAILABLE FROM: HTTPS://EZPROXY.UOW.EDU.AU/LOGIN?URL=HTTPS://SEARCH.EBSCOHOST.COM/LOGIN.ASPX?DIRECT=TRUE&DB=RZH&AN=133645941&SITE=EHOST-LIVE	Ineligible study design
WALSH AL. NURSE RESIDENCY PROGRAMS AND THE BENEFITS FOR NEW GRADUATE NURSES. PEDIATRIC NURSING [INTERNET]. 2018; 44 (6):275–9. AVAILABLE FROM: HTTPS://EZPROXY.UOW.EDU.AU/LOGIN?URL=HTTPS://SEARCH.EBSCOHOST.COM/LOGIN.ASPX?DIRECT=TRUE&DB=RZH&AN=133645941&SITE=EHOST-LIVE	Ineligible study design
WARREN JI, PERKINS S, GREENE MA. ADVANCING NEW NURSE GRADUATE EDUCATION THROUGH IMPLEMENTATION OF STATEWIDE, STANDARDIZED NURSE RESIDENCY PROGRAMS. JOURNAL OF NURSING REGULATION [INTERNET]. 2018; 8 (4):14–21. AVAILABLE FROM: HTTPS://EZPROXY.UOW.EDU.AU/LOGIN?URL=HTTPS://SEARCH.EBSCOHOST.COM/LOGIN.ASPX?DIRECT=TRUE&DB=RZH&AN=127421618&SITE=EHOST-LIVE	Ineligible study design
WOLFORD J, HAMPTON D, THARP-BARRIE K, GOSS C. ESTABLISHING A NURSE RESIDENCY PROGRAM TO BOOST NEW GRADUATE NURSE RETENTION. NURSING MANAGEMENT [INTERNET]. 2019; 50 (3):44–9. AVAILABLE FROM: HTTPS://EZPROXY.UOW.EDU.AU/LOGIN?URL=HTTPS://SEARCH.EBSCOHOST.COM/LOGIN.ASPX?DIRECT=TRUE&DB=MNH&AN=30817432&SITE=EHOST-LIVE	Duplicate study
WOLFORD J. ESTABLISHING A NURSE RESIDENCY PROGRAM TO BOOST NEW GRADUATE NURSE RETENTION. NURSING MANAGEMENT [INTERNET]. 2019; 50 (3):43–9. AVAILABLE FROM: HTTPS://EZPROXY.UOW.EDU.AU/LOGIN?URL=HTTPS://SEARCH.EBSCOHOST.COM/LOGIN.ASPX?DIRECT=TRUE&DB=A9H&AN=134820357&SITE=EHOST-LIVE	Duplicate study

**Table 7:** Included studies.

Included Studies
• Cadmus, E. and Roberts, M. First Year Outcomes: Program Evaluation of a Statewide Nurse Residency Program. (2022) JONA: The Journal of Nursing Administration, 52 (12).
• Findings from Sharp HealthCare in the Area of Nursing Administration Described (A 1-year Accredited Nurse Residency Program's Effect on Intent to Leave). Health & Medicine Week [Internet]. 2021 Dec 24 [cited 2023 Apr 1]; 2178. Available from: https://search.ebscohost.com/login.aspx?direct=true&AuthType=sso&db=edsgih&AN=edsgcl.687356678&site=eds-live&scope=site
• Miller CM, Meyer K, Riemann LA, Carter BT, Brant JM. Transition into Practice: Outcomes of a Nurse Residency Program. The Journal of Continuing Education in Nursing [Internet]. 2023 Jan 1 [cited 2023 Apr 1]; 54 (1):32. Available from: https://search.ebscohost.com/login.aspx?direct=true&AuthType=sso&db=edsgih&AN=edsgcl.732686211&site=eds-live&scope=site
• Salmond SW, Cadmus E, Black KK, Bohnarczyk N, Hassler L. Long-Term Care Nurse Residency Program: Evaluation of New Nurse Experiences and Lessons Learned. The Journal of continuing education in nursing. 2017; 48 (10):474–84. Available from: https://search.ebscohost.com/login.aspx?direct=true&AuthType=sso&db=edsghw&AN=edsgcl.514226799&site=eds-live&scope=site
• Wolford, J., Hampton, D., Tharp-Barrie, K., & Goss, C. Establishing a nurse residency program to boost new graduate nurse retention. (2019). Nursing management, 50 (3), 44–49. https://doi. org/10.1097/01.NUMA.0000553497.40156.4e

Data were extracted from the included and eligible studies using the JBI extraction tool. The extracted data included details such as the authors, year of publication, study design, setting and participants, methods and interventions, and outcomes. The result of the data extraction were displayed in a tabulated format (Table 3-a, Table 3-b, Table 3-ca, 3b, and 3c).

### 2.7 Data synthesis

All included studies were critically appraised by the two researchers using the JBI tools based on the study type. Any study that lacked the robustly method was excluded, and the number of all excluded studies are clearly mentioned in the PRISMA flow chart along with the exclusion reason (Fig. 1). The extracted data showed heterogeneity in the studies methodologies, however, homogeneity in the study outcomes. Therefore, study outcomes were pooled, as the programs outcome was similar which was the retention of the nurses who completed the nurse residency program. This was followed by discussion of the results and exploring relationships within studies and assessing the robustness of the synthesis and effect size.

## 3 Results

### 3.1 Study inclusion

From the electronic databases identified in the methodology, 189 studies met the inclusion and exclusion criteria. There were 48 duplicate studies removed before importing the articles to JBI Sumari. The researchers excluded 104 references after screening titles and abstracts and retrieved 37 studies for full-text evaluation, of which 7 studies met the inclusion criteria and were included in the review. After critical appraisal of the studies, five were included (Table 7). During the full text screening, studies were excluded due to being a duplicate, ineligible outcomes, ineligible study design, ineligible phenomena of interest, student thesis, newsletter editorial, outside the range of study dates.

During the critical appraisal, two studies were excluded as they had either a sample size is small which affected the credibility of the results or program evaluation and not a study. One study was secondary data analysis, these types of studies are considered weak, and they were unable to retrieve all data, therefore, it was excluded. The final total number of studies included were five. Refer to the PRISMA flow chart (Fig. 1).

The review included studies were all conducted in the United States. Study designs were Experimental retrospective pre-post-test, prospective, longitudinal, quasi-experimental study, Analytical Cross-Sectional Study, Longitudinal Prospective Study-Case series, and mixed-methods study.

### 3.2 Study characteristics and risk of bias

All identified studies were assessed by two researchers (ZM & NA) against the Cochrane Risk of Bias in Non – randomized studies of intervention (ROBINS-I) tool [25]. The studies ranged between moderate to low risk based on the critical appraisal (Table 4-a, Table 4-b, Table 4-c, Table 4-dd). Interestingly, among all studies the mean age range was 28.5 years. In addition, there were differences in the level of education of the participants and there was no control on those confounding factors. Salmond et al., 2017, had difficulty in collecting data for different reasons such as; participants lack of experience in completing surveys, survey fatigue, lack of Information Technology support, two types of data on soft copies and hard copies and finally, several facilities were not able to provide the data on retention to the researchers [11]. However, the researchers tried to have the study as mixed methodology to support as the most effective mean of putting data into context, which has led to considering the overall risk for bias as a moderate risk.

### 3.3 Results of individual studies

Miller et al., 2023 carried out a prospective, longitudinal, quasi-experimental investigation in 2023 in a fully integrated, three-time Magnet Hospital [13]. Seven cohorts with total 106 freshly hired graduate nurses, were enrolled in this trial and assigned to one of two-study arms [13]. Either a nurse residency intervention group or a control group receiving regular nursing orientation [13]. Nurse resident = 77, control group = 29. The retention rate of nurse residents was 77.27% for the three cohorts enrolled in 2016.90.20% for the three cohorts enrolled in 2017. Moreover, 89.71% for the cohort enrolled in 2018. Casey-Fink Surveys measuring group outcomes included Readiness for Practice, Nurse Retention, and Nurse Work Satisfaction. The study concluded that compared to normal orientation, the NRP led to increased job satisfaction, nurse retention, and preparation for practice. Research has shown that when new nurses demonstrate high job satisfaction, competence, and confidence in their preparation they tend to have higher retention rates. Importantly, the characteristics of ANCC accredited programs of Nurse Residency programs, which is a mandate for obtaining Magnet recognition, have several domains such as mentorship, preceptorship, wellbeing programs, Quality & safety educations such as the QSEN.

In another study conducted by Failla et al., 2021 which was a longitudinal prospective study they demonstrated that the 117 nurse graduates who completed Nurse residency program had a Retention rate of 85% at 12 months. In addition, they reported that since the inception of the system NRP, the 1-year retention is greater than 96% (n = 241) [12]. They have concluded that Residency Programs and resources can be used by nurse leaders to enable newly hired graduate nurses feel empowered and engagement with the organization and may guarantee that development of professional nurses who are actively immersed, competent, and clinically proficient [12]. Those factors shall positively influence the retention rate of new nurses. In the same study, with the intention of leaving as the dependent variable and considering all other study variables as independent or predictor variables, a multiple regression analysis was carried out and were found to be statistically significant, (P = 0.000). This result indicates that when supporting the Nurse residency programs is decreased, the intent to leave increases leading to reduced retentions. This might explain the drop noticed in the retention rates of the second year and forward in comparison to the retention rates of the first year among several studies.

Interestingly, when the residency program was tested utilizing a pertest–post-test, mixed-methods approach study in 36 long term facilities by Salmond et al. (2017) with the study's objective of evaluating the residency program influence on new nurses' perceptions of organizational safety, competence, retention, and job satisfaction, they concluded that no matter the setting, through the first 12 months, the NRP had an 86% retention rate. Since this study was a mixed methodology, they utilized the Casey Fink Graduate Nurse Experience Survey tool as well as focus group interview and they both supported the conclusion that new nurses thought that the residency program gave them the opportunity to develop their confidence and skills in caring for the patients as well as responding to queries. In addition, results from the Preceptorship Program Assessment Tool also supported the enhanced competence and confidence, with 100% of respondents reporting that the program boosted their awareness of LTC rules and procedures and raised their confidence in making decisions regarding managing care. Overall, satisfaction, increased confidence, and competence as well as retention rates were concluded by the study despite the expressed limitation of the program lacking nurse residents' managers, colleagues and the inconsistent preceptor support. All of those imply the importance of having such programs supported by the Nursing leadership. Finally, there was a selection biased as the nurse residency program was new, and limited numbers participated, and there was no randomization for being part of the control or intervention group. They reported it was seen as recipients of favouritism.

Pillai et al., 2018 conducted a quasi-experimental study with the purpose of examining hiring and tracking data of registered nurses (RNs) to determine the 1-year retention rate and ongoing retention of GEMS. They had 241-nurse residents who had 14 weeks long home-grown nurse residency program in a tertiary hospital. They obtained 212 nurses retained after one year with retention rate of 96%. The GEMS home-grown program retention rates were higher than vendor-based programs. Healthcare organizations may find it challenging to obtain and utilize company-based software that support Nurse Residency programs with the educational content and competency check needed such as like Versant's Competency-Based System. It would be challenging to generalize the ideal program structure and content because of the additional funding it would bring to the program. Interestingly organizations that has created their own in-house programs had the excellent retention rates as well.

Wolford et al., 2019 conducted an experimental retrospective pre-post-test at Healthcare system comprised of five hospitals with Control group = 791 newly licensed nurses hired prior to the NRP and an intervention group = 232 newly licensed nurses enrolled in a nurse residency program [14]. There was a significant decrease in turnover for the group who participated in the NRP. 14% of turnover among the control group with a retention rate of = 86%, compared to 3.5% of turnover from intervention group [14]. Wolford et al., 2019 estimated that $44,085.61 was made for the organization's replacement cost of a newly licensed RN. To offset the cost of orientation, it was found that an NLRN must work 1198 h, or 8.32 months (36 h/week), in a productive status. The total cost of the NRP during the research period was $97,777, which included the manager's salary, supplies, paperwork, parking, and catering expenses [14]. Preventing the turnover of three NLRNs during their first 8.32 months of employment in a productive state is the program's cost break-even point [14].

Nurse residents' readiness for practice and opinions of nurse retention both considerably increased, suggesting that nurse residents were more likely to be kept at the organization. Moreover, a distinguishing hallmark of hospitals earning Magnet designation is transformational leadership, which has been recommended to support new nurses during the transition phase and the nurse residency programs. The American Nurses Association recognizes the high-quality care that hospitals provide through the developed Magnet Recognition Program through the American Nurses Credentialing Center (ANCC) [9]. Hospitals with the designation of “Magnet Hospital” are distinguished for their healthy work environments and outstanding support of nurses' wellbeing and high retention rates of nurses due to the outcome driven nature and the transformational leadership [9].

## 4 Discussion

The existing literature has consistently shown a significant decline in new nurses’ turnover rates with the Nurse Residency Programs. In addition, it has accompanied retention with notable supplementary effects such as amplified commitment, self-assurance, competence, and engagement among the nurses who participated nursing residency program. The imperative of justifying the cost of Nurse Residency Programs has necessitated that executive leadership and administrative directors of the program seek compelling evidence of return on investment. Such a demand has imposed tremendous pressure on Chief Nursing Officers (CNOs), Program Directors and Professional Development leaders. Consequently, healthcare leaders demand expedited and tangible outcomes, making the allocation of resources to professional development a daunting and delicate undertaking. Nevertheless, Nurse Residency program directors ought to be capable of articulating the short- and long-term programmatic goals to satisfy sponsors and stakeholders' anxieties regarding the programs' efficacy and durability.

Cost is the main reason given for not implementing nurse residency programs [20]. Residency programs are expensive, whether one the organization opts for purchasing an existing program model or creates and staffs their own [20]. The irony is that the cost of not implementing such programs is also very high since that would mean nurses shortage, interrupted patient care, drop in patient satisfaction, cost of hiring and recruiting new nurses along with the cost of orientation and training for the new hire [20]. None of the identified studies discussed the cost of the program they had whether they were vendor based or home grown to understand the cost difference and return of investment with the Nurse Residency programs.

In any healthcare institution, nurses’ shortage causes a huge workforce volatility that influences high cost, impacted quality of care, and patient and staff satisfaction. The cost to the organization is made up of recruitment expenses which include costs associated with recruiting, interviewing, training, and backfilling. According to reports, it would cost between $40,0385 and $88,000 to replace one nurse. In addition, a 1% increase in turnover in an organization is thought to cost $300,000 a year. Future of Nursing report 2020–2030 by the National Academy of Medicine recommended that Healthcare organizations develop and revaluate nurse transition programs such as the nurse residency programs that help new nurses adjust to their new roles [16].

Nursing Residency Programs (NRPs) appear to have a positive impact on lowering first-year turnover rates. However, retention rates decrease slightly during the second year, and there is currently a lack of data on retention rates beyond this point. It is important to address potential confounding factors and conduct further studies to explore the reasons behind this decline in retention rates after the first year, which may relate to the absence of continuous educational support provided during the residency program, differences in the level of education and nursing degree upon starting the nurse residency program. Additionally, other factors such as nurse immigration should be taken into consideration.

One of the most significant and long-standing phenomena is the movement of the medical workforce around the world. This phenomenon is linked to the so-called medical “brain drain,” which typically occurs from less developed nations to societies with greater economic prosperity. For example, in February 2023, New Zealand offered overseas nurses and midwives an immediate pathway to residency, as the country tries to address an acute shortfall of health [15]. The government has changed immigration system so that well trained nurses from other countries who are eligible get fast-tracked residency [15]. Despite the extensive investments and well-structured transition to practice programs, the “brain drain” might impact the retention after the first year [15].

The American Nurses Credentialing Center (ANCC) suggests that there is a need for Transition Programs, not only for newly graduated nurses, but also for those undergoing a change in specialty or work settings, such as through fellowship programs [23]. There is a lack of research on Transition to Practice fellowship programs and programs targeting nurses who change specialties within the same hospital. For instance, nurses transferred or reshuffled from medical units to critical care units do not have structured transition plans. Given the fast-paced, dynamic nature of hospitals such transitions are not supported well. However, the American Association of Colleges of Nursing (AACN) (US) heavily supported the adoption of NRPs in the US [24]. They believe that these programs will smooth the transition through a number of methods, such as boosting competence and confidence and enhancing retention [24].

The ANCC PTAP accreditation mandates five domains for accrediting Nurse Residency programs, ensuring high-quality outcomes. The latest PTAP updates of 2023 mandates identifying quality outcomes that measures then patient care outcomes and the impact of the nurse residency program on patient quality of care. A standardized program structure would assist in assessing and validating program effectiveness and enabling replication of results across different contexts and countries. The standardization of Nurse Residency programs is particularly important within a single state or country, given the variability in approaches implemented which can make outcome comparisons difficult. However, such programs must be tailored to the unique needs, mission and vision of the organization, as well as the context and environment in which they operate. The development of a state-wide residency model represents a novel structure that requires continual assessment and adaptation. As part such initiative, the regulatory bodies shall collaborate with clinical coordinators and directors at each facility to design their 12-month plans for residency objectives, goals, curriculum design and content, application procedures and evaluation methods. This shall support standardization and would require periodical assessment, quantitative and qualitative outcomes as well as up to 5 years retention rates.

While being largely implemented throughout Western countries, NRPs have gained recognition in other nations. For instance, In Jordan the new graduate nurses orientation programs range from six weeks to three months, to familiarize new nurses with policies and procedures, however, Jordanian authorities have started exploring into the advantages and difficulties of deploying NRPs. Having Magnet accredited hospitals in Jordan has been an exemplary of excellence and new nurses’ retention where having a structured NRP is one of the criteria for the accreditation. Entry-to-practice programs were also effectively adopted in New Zealand hospitals, which reported a good impact on turnover, and improvement in new nurse graduates work satisfaction. The naming is different in New Zealand however, the concept, structure and expected outcomes are similar to those of the NRP [11]. More than a two-year span, participating facilities raised NGN employment retention rates to 80% in New Zealand ^11.^ The establishment and adoption of a national standard format for NRPs could boost their success on a national level which might be the solution to avoid the brain drain [17].

Other factors support the Nurse Residency program such as having incremental orientation checklist that allows new nurses to learn at their own pace. Having trained Preceptors is of a paramount importance in the organization to support a smooth transition of the nurse residents and help enculturating and assimilating them to the culture and the profession. In addition, availability of mentors and other support system in the hospital such as counselling office, wellbeing council and resources add to the success of the nurse residency program. Preparation, support, and recognition are important for the leader to acknowledge. The new generation joining the nursing profession and the millennials need to be kept engaged and involved in their learning process. Finally, another important topic to consider which is an unfortunate finding by an integrated review conducted by D'ambra & Andrews, 2013 was the Incivility that the new nurses spoke of [18]. It is important to deal with incivility among nurses through transformational organizational leadership that promotes respectful work cultures in which nursing staff are offered chances for development, education and support from qualified mentors and preceptors [18]. Healthy work settings, according to Kramer, Brewer, and Maguire (2011), foster good communication, encourage Interprofessional care, and provide a learning environment [19]. According to Hopkins et al., 2016, nursing residents who participate in NRP exhibit effective and assured Interprofessional communication, which has improved [21]. This is in line with Salmond et al., 2017 claim that at 3-month of the NRP program's completion graduates' capacity to seek assistance boosted [22].

## 5 Conclusion

The purpose of this systematic review was to evaluate the effectiveness of Nurse Residency programs in improving the new nurses' retention rates. Nurse residency programs are proven to be great educational intervention to support with the nurses’ shortage and develop well supported, competent and confident new nurses. It is recommended to conduct a proper RCT to measure the effects of such programs on the nurse retention.

Possible areas for further investigation include analysis of nationwide programs in the UAE that are mandated by regulatory bodies. In addition, understanding the real reasons behind the drop of the retention achieved in the first year and develop programs that can improve retentions for more than 12 months.

## 6 Recommendations

### 6.1 Recommendations for practice or policy in the UAE

The results of this systematic review emphasize the importance and effectiveness of Nurse Residency Programs in retaining new nurses. In the context of the United Arab Emirates (UAE), it is strongly advised to establish structured Nurse Residency Programs with support from organizational policies and nursing leadership. These programs should be developed by nursing education, with clearly defined quality standards and performance indicators to measure retention rates, the satisfaction of new registered nurses, and their confidence levels. In light of the systematic review findings, the following policy guidelines are recommended for healthcare facilities implementing nurse residency programs in the UAE:

• Nurse residents should be assigned dedicated and well-trained preceptors throughout the program, as this was associated with higher retention rates in the analyzed programs.
• All nurse residency programs should have a minimum duration of 12 months to improve retention rates.
• The nurse residency program should be integrated into the organization's strategic plan as a workforce development and retention strategy, with continuous evaluation of the program's effectiveness, nurse residents' confidence, satisfaction, and retention rates.

### 6.2 Recommendations for research in the UAE

This systematic review highlights the need for rigorous research that goes beyond program evaluations. In the UAE, as the structures for nurse residency programs are standardized, future research should be conducted to produce conclusive and statistically significant findings that can be generalized. There is a scarcity of studies on nurse residency programs in the Middle East and Gulf countries, which heavily rely on an expatriate nursing workforce. Future studies should incorporate larger sample sizes and address confounding factors through discussion and control to enhance the reliability of their findings.

## Declaration of using artificial intelligence

The authors utilized AI for paraphrasing certain paragraphs to enhance clarity, and this was carried out in response to the revision request.

## Data availability statement

The authors confirm that the data supporting the findings of this study are available within the article [and/or] its supplementary materials.

## CRediT authorship contribution statement

**Zahra Mohamed:** Writing – original draft, Formal analysis, Conceptualization. **Nathira Al-Hmaimat:** Writing – review & editing, Supervision, Formal analysis.

## Declaration of competing interest

The authors declare that they have no known competing financial interests or personal relationships that could have appeared to influence the work reported in this paper.
